# The Role of Senescence in Experimental Periodontitis at the Causal Level: An in Vivo Study

**DOI:** 10.3390/cells14030226

**Published:** 2025-02-05

**Authors:** Xiaogang Chu, Mahmoud Elashiry, Angelica Carroll, Celine Joyce Cornelius Timothius, Christopher W. Cutler, Ranya Elsayed

**Affiliations:** Department of Periodontics, Dental College of Georgia, Augusta University, Augusta, GA 30912, USA; xichu@augusta.edu (X.C.); melashiry@augusta.edu (M.E.); ancarroll@augusta.edu (A.C.); ccorneliustimot@augusta.edu (C.J.C.T.); chcutler@augusta.edu (C.W.C.)

**Keywords:** periodontal disease, periodontitis, senescence, P16-3MR, bone loss

## Abstract

The occurrence and severity of periodontitis (PD) tend to increase with age, and yet the underlying mechanisms remain unclear. Immune senescence is known to be triggered in mice and humans as they age. Experimental PD in mice has been shown to induce senescence biomarkers p16 ^INK4a^ and p21, dysfunction of antigen-presenting cells (APCs), and activation of the senescence-associated secretory phenotype (SASP). However, the causal links of senescence to experimental PD are not yet established. This study aims to elucidate the role of senescence in experimental PD at a causal level. The P16-3MR mouse model harbors the p16^INK4a^ (Cdkn2a) promoter, driving in vivo expression of synthetic Renilla luciferase, monomeric red fluorescent protein (mRFP), and herpes simplex virus-1 thymidine kinase (HSV-TK). This facilitates in vivo identification of p16 ^INK4a^ activation at the cellular level and the consequences of selective elimination of p16^INK4a^-positive cells by ganciclovir (GCV) treatment. Mice were treated with/without GCV for two weeks during ligature-induced PD. In vivo bioluminescence imaging quantified p16^INK4a^ activation, while Western blot and immunofluorescence analyses assessed key senescence and inflammatory markers (p16, p21, p53, Cyclin D1, p-H2A.X, IL17, and IL1β). Alveolar bone volume was analyzed by micro-CT and histomorphometry. Our findings demonstrate that clearance of senescent cells in mice subjected to experimental PD alleviates inflammation and mitigates bone loss. These results suggest a causal role for senescence in PD pathology, raising the future prospect of senolytic agents for therapeutic intervention in PD.

## 1. Introduction

Periodontitis (PD) is a chronic inflammatory disease that impacts tissues surrounding and supporting teeth and is closely associated with aging [1,2]. In the U.S., the prevalence of PD is 47.2% in adults over 30, which increases to 70.1% in those 65 and older [3]. The prevalence and severity of PD are significantly higher in older people compared with young people [4,5]. As individuals age, they become more susceptible to PD due to immune function changes, oral microbiome composition, systemic disease, and overall health status decline [6,7].

While it is widely accepted that periodontitis is primarily initiated by bacterial infection, resulting in inflammatory destruction of the tooth-supporting alveolar bone and soft tissue [8], the relationship between aging and PD is unclear. Cellular senescence, a fundamental mechanism of aging, plays a critical role in various age-related diseases [9]. Initially described as the limited proliferation capacity of normal human cells in vitro, cellular senescence is now recognized as a multifaceted biological process with significant implications for tissue physiology and pathology [10]. Senescent cells accumulate in various tissues with aging or stress and release soluble or extracellular vesicle (EVs)-bound pro-inflammatory cytokines, chemokines, and proteases, collectively known as the senescence-associated secretory phenotype (SASP). SASP can severely impair tissue function and has been implicated in the progression of numerous age-related disorders. Consequently, therapeutic strategies targeting the selective elimination of senescent cells or attenuating SASP factors have emerged as promising avenues for mitigating age-associated diseases [9,11]. Cellular senescence can be induced through two primary pathways: replicative senescence and stress-induced senescence. The latter is triggered by various internal or external stressors, including traumatic injury, oxidative stress, radiation, or chemotherapy [12]. These stressors cause cells to rapidly accumulate DNA damage, leading to activation of the DNA damage response. Key mediators of cellular senescence include p53, p21, and p16, which inhibit Cyclin-dependent kinases, leading to cell cycle arrest and induction of cellular senescence.

Our group previously demonstrated that Porphyromonas gingivalis (Pg), a keystone pathogen in PD pathogenesis, can infect dendritic cells (DCs) and induce DC senescence [13]. Pg infection was found to induce the activation of SASP exosomes (EXOs), nano-sized EVs, from DCs. In addition, Pg-induced SASP EXOs transmitted cellular senescence to bystander cells in periodontal tissue and accelerated bone degeneration in mice [14]. While periodontitis (PD) is known to be a disease associated with inflammaging, the underlying molecular and cellular mechanisms remain poorly understood. Age-related transcriptional and functional changes in gingival cells in response to microbial challenges have been documented, but their role in PD remains unclear [15]. Cause-and-effect studies in experimental PD models are needed to elucidate the roles of senescence and the SASP in PD and to identify the principal inflammatory cells and stressors involved. A comprehensive understanding of the complex interplay between aging, cellular senescence, and periodontitis is crucial for developing effective strategies in the prevention and treatment of periodontal disease. The present study employed p16-3MR mice with experimental PD to track and selectively eliminate senescent cells, demonstrating the role of cellular senescence in PD at the causal level.

## 2. Materials and Methods

### 2.1. Ethics Statement

All experimental procedures were approved by the Institutional Animal Care and Use Committee (IACUC) of Augusta University under protocol (2022-1073, approval date: 20221215). The protocol ensures compliance with ethical standards for animal research. All animal experiments were conducted following the guidelines established by the National Institutes of Health (NIH).

### 2.2. P16-3MR Mice

**Figure 1:**
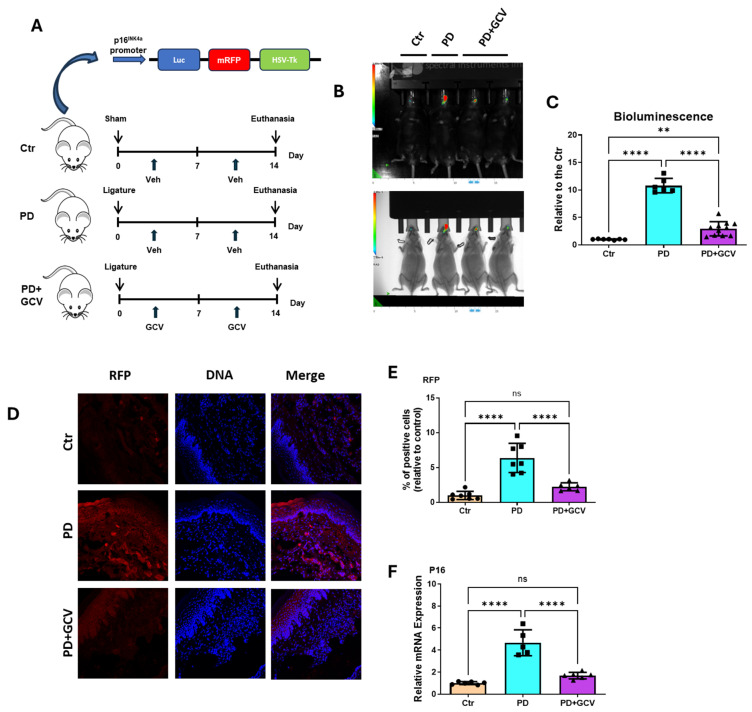
(**A**) Animal study design. p16-3MR mice were randomly divided into three groups (N = 8–10/group). Group 1 (Ctr) had no ligature, Group 2 (PD) was subjected to ligature-induced PD (LIPD), Group 3 (PD+GCV) had LIPD and GCV injection. Ganciclovir (25 mg/kg in PBS) was administered via daily intraperitoneal (i.p.) injections for 5 consecutive days per cycle, with two cycles in total. Grps 1 and 2 received equal volumes of PBS injections. (**B**) In vivo live imaging of luminescence in control, PD and PD+GCV mice. Different groups were locally injected with coelenterazine in the ligature area, and luminescence was quantified using an IVIS spectrum in vivo imaging system. (**C**) Quantification of luminescence in the three groups (N = 6–12 each group). (**D**) Gingival tissues of different groups of p16-3MR mice were collected 14 days after treatment, fixed, and stained for nuclei (Hoechst; blue) and RFP (immunostaining; red) antibody. (**E**) Quantitation of the RFP using Image J software (NIH) and plotted as relative fold change in positive percentage. (N = 6–7/group). (**F**) Relative mRNA expression of P16 in gingival tissues of 3 groups of P16-3MR mice. ** indicates *p* < 0.01, **** indicates *p* < 0.0001, and ns indicates *p* > 0.05.

P16-3MR original breeding pairs were generously provided by Dr. Judith Campisi’s lab. The mouse p16^INK4a^ promoter drives the expression of the 3MR (trimodality reporter) fusion protein, which includes functional domains of a synthetic Renilla luciferase (LUC), monomeric red fluorescent protein (mRFP), and truncated herpes simplex virus 1 (HSV-1) thymidine kinase (HSV-TK) [16,17]. (Figure 1A). The expression of P16 is upregulated in cellular senescence. Synthetic Renilla luciferase (LUC) and monomeric red fluorescent protein (mRFP) enable bioluminescent and fluorescent P16-positive senescent cell imaging, respectively. In cells expressing HSV-TK, ganciclovir (GCV) is converted to an active form that kills non-dividing senescent cells through mitochondrial DNA (mtDNA) damage and apoptosis [18]. This process selectively targets p16^INK4a^-expressing senescent cells without affecting non-senescent cells. Genotyping was accomplished by PCR using primer pairs spanning the transgenic region. The primers (Invitrogen, Waltham, MA, USA) used for the genotyping are shown in Supplementary Table S1. The PCR conditions included an initial 5-min denaturation at 95 °C, followed by 35 cycles of 30 s at 95 °C, 30 s at 55 °C, and 60 s at 72 °C, with a final 7-min extension at 72 °C. Only p16-3MR-positive mice were used to ensure homogeneity. p16-3MR-positive and -negative mice were distinguished using PCR. Mice with bands corresponding to the transgene (two bands) are considered p16-3MR-positive (Supplementary Figure S1). Animals that met the required health conditions were included in the study, while those displaying signs of illness or abnormalities during the acclimatization period or during the treatment phase were excluded. Animals that lost their ligatures at the time of the tissue harvest were excluded from the study.

### 2.3. Induction of Experimental Periodontitis (PD) in Mice Model

The PD mice model was developed by ligating the upper right second molar with a black silk suture to induce inflammation and alveolar bone loss, as described previously [14,19]. In this study, young male P16-3MR mice, aged between 2 and 4 months, were subjected to the placement of a ligature on the maxillary second molar utilizing a silk 5–0 suture. The ligature was retained for the entire duration of the experimental period, which spanned 14 days. The contralateral molar in each subject was left un-ligated to serve as a baseline internal control for the assessment of alveolar bone volume. The mice were randomly divided into three groups: control, PD, and PD treated with GCV (N = 10 for each group). For each experiment, at least five mice were placed in each group (α = 0.05, β = 0.8) [20]. Allocation was concealed during the randomization process. Blinded analysis was performed; animals/samples were coded during data collection and analysis.

### 2.4. Ganciclovir (GCV) Treatment

A fresh stock solution of ganciclovir (GCV) was prepared at 2 mg/mL in PBS. GCV was administered through daily intraperitoneal (i.p.) injections, given 5 days per week, at a dosage of 25 mg/kg in PBS, as documented in previous reports [16]. Control and PD mice groups were injected with an equal volume of PBS.

### 2.5. P16-3MR Mice Bioluminescence Imaging

The in vivo imaging system (IVIS) quantified bioluminescent signals in photons per second, displaying them as intensity maps. Luminescence, generated by photon flux from luciferase-expressing cells, was measured at the injection site. Animals were administered 5–10 μL of h-Coelenterazine solution (0.5 mg/mL in distilled water) orally or subcutaneously, 30 min prior to imaging. Mice were anesthetized using an isoflurane vaporizer and positioned inside the IVIS spectrum imager’s camera box. Bioluminescence imaging was performed, followed by X-ray imaging to provide anatomical reference and overlay the bioluminescent signals onto the skeletal structures.

### 2.6. qPCR

Total RNA was extracted with the QIAGEN RNeasy Mini Kit (Cat#: 74134, Qiagen, Inc., Valencia, CA, USA). RNA concentration and purity were evaluated using a NanoDrop 1000 UV-VIS Spectrophotometer (Thermo Fisher Scientific, Software Ver.3.8.1), with a 260/280 ratio of 2.0 deemed acceptable for further analysis. Reverse transcription to cDNA was conducted in a 20 µL reaction volume using the High-Capacity Reverse Transcription Kit (Applied Biosystems, Thermo Fisher Scientific, Waltham, MA, USA). Quantitative real-time PCR was carried out with SYBR Green qPCR Master Mix (Applied Biosystems, Thermo Fisher Scientific, Waltham, MA, USA), and relative gene expression was calculated using the delta-delta CT method and plotted as fold changes. The primers used for qPCR are listed in Supplementary Table S2.

### 2.7. Immunoblotting Analysis

Immunoblotting was performed as previously described [21]. Briefly, tissues were homogenized in extraction buffer (RIPA buffer, Thermo Scientific, Waltham, MA, USA), and lysates were clarified by centrifugation. Protein concentrations were measured using the Bradford assay (Bio-Rad Laboratories, Hercules, CA). Equal amounts of protein were separated on 8–15% SDS–PAGE, transferred to polyvinylidene difluoride (PVDF) membranes, and incubated with specific primary antibodies overnight at 4 degrees followed by horseradish peroxidase-conjugated secondary antibodies (Cell Signaling). Signal intensities were detected using standard methods and analyzed using Image J software (ImageJ 1.54g). Details of the primary antibodies used are provided in Supplementary Table S3.

### 2.8. Histologic Examination

Mouse gingival tissues were dissected and fixed in 4% paraformaldehyde (PFA). Fixed tissues were dehydrated in a graded ethanol series, embedded in paraffin blocks, sectioned, and mounted on glass slides. Hematoxylin and eosin (H&E) and immunofluorescence staining were performed using standard methods. Briefly, animal tissue sections were subjected to antigen retrieval followed by blocking with 2.5% bovine serum albumin in PBS. Sections were incubated with primary antibodies overnight at 4 °C followed by washing and incubation with the DNA stain Hoechst and Alexa Fluor 488– or 594–conjugated secondary antibodies (1:1000 goat anti-rabbit, anti-mouse, or anti-rat, Invitrogen, Waltham MA). An anti-fade kit (Invitrogen, Waltham, MA, USA) was applied to prevent photobleaching. Images were acquired using a Zeiss 780 Upright Confocal microscope (Carl Zeiss) and processed using standard methods. Fluorescence intensity was quantified using ImageJ and plotted as relative fold change. Details of the primary antibodies are listed in Supplementary Table S4.

### 2.9. Micro-CT Imaging and Bone Parameter Analysis

The maxillae were scanned using a 1272 Skyscan System (Bruker, Belgium). Three-dimensional reconstruction was performed using NRecon 1276 software (Skyscan), and alveolar bone loss around the ligated maxillary second molar was analyzed using CTAn software v.1.18 (Skyscan), as previously described [14]. Scanning parameters were set at 9.5 μm image pixel size with a 0.25 mm Al filter, 0.2 rotation step, and frame averaging of 4. The region of interest (ROI) was standardized across all samples, starting at the cementoenamel junction of the mesial surface of the second molar and extending to the root apex (40 slices) in the occluso-apical direction. The mesio-distal direction was defined from the distal end of the maxillary first molar to the mesial end of the maxillary third molar. Bone volume (BV, μm^3^) around the second molars was quantified, and 3-D models were generated, including teeth. The un-ligated contralateral molar was an internal control for alveolar bone volume quantification [14].

### 2.10. Leukocyte Acid Phosphatase (TRAP) Staining

The tissues were fixed using formalin. Mineralized bone was decalcified using Ethylenediaminetetraacetic acid (EDTA). Trap stainings were performed according to the manufacturer’s protocol (Sigma-Aldrich 387A).

### 2.11. Statistical Analysis

Data analysis was conducted using GraphPad Prism 10.3.1 (GraphPad Software, La Jolla, CA, USA). Statistical tests included two-way ANOVA, one-way ANOVA, or Student’s *t*-test, with significance defined as *p* < 0.05 and a 95% confidence interval. Post hoc multiple comparisons were performed using Tukey’s or Bonferroni tests. The Shapiro–Wilk test was used to evaluate the assumption of normality. If normality was not met, appropriate non-parametric tests were applied. The results are presented as mean ± standard deviation (SD). Outliers identified through statistical analysis (beyond three standard deviations) were excluded.

## 3. Results

### 3.1. Periodontitis (PD) Induces P16 Expression, Inhibited with Ganciclovir (GCV) Treatment in p16-3MR Mice Model

The p16-3MR transgenic mouse model provides a powerful tool for studying cellular senescence in living animals [16,17]. Using this model, we can track, utilizing luminescent or fluorescent imaging, and selectively eliminate, with GCV treatment, senescent cells, providing valuable insights into the role of cellular senescence in various physiological and pathological processes.

**Figure 2:**
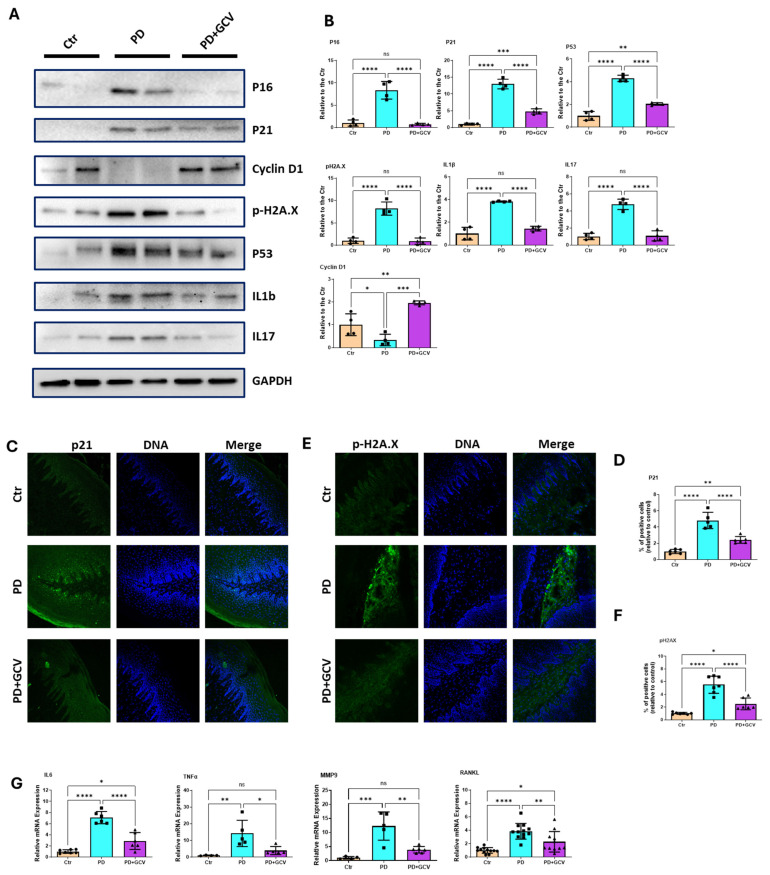
(**A**) Representative immunoblotting of p16, p21, p53, Cyclin D1, p-H2A.X, IL17, and IL1β in gingiva tissue. GAPDH was used as the loading control. (**B**) Densitometric analysis of p16, p21, p53, Cyclin D1, p-H2A.X, IL17, and IL1β protein expression in gingival tissues using Image J software (NIH), N = 4 each group. (**C**) Representative immunofluorescence staining of p21in gingival tissue. Nuclei were stained by Hoechst. (**D**) Quantitation of the p21 using Image J software (NIH) was plotted as a relative fold change in the percentage of positive cells. N = 6–7/group. (**E**) Representative immunofluorescence staining of p-H2A.X in gingiva tissue. Nuclei were stained by Hoechst. (**F**) Quantitation of the p-H2A.X using Image J software (NIH) and plotted as a relative fold change in the percentage of positive cells. N = 6– 7/group. (**G**) SYBR green real-time PCR amplification of IL-6, TNF-α, MMP9, and RANKL from gingiva tissue. The results shown represent mean ± SEM for 6-7 animals in each group, with two replicates. * indicates *p* < 0.05, ** indicates *p* < 0.01, *** indicates *p* < 0.001, **** indicates *p* < 0.0001, and ns indicates *p* > 0.05.

In the present study, p16-3MR mice were randomly divided into three groups (N = 8). Group 1 (Ctr) served as a baseline control with no ligature and received a sham injection with PBS. Group 2 (PD) received ligature and a sham injection with PBS. Group 3 (PD+GCV) received ligature with GCV injection (Figure 1A). At the conclusion of the experimental period, the substrate, h-Coelenterazine, was administered locally, and the luminescence intensity was quantified by in vivo bioluminescence. As depicted in Figure 1B,C, the luminescence intensity was notably heightened in PD mice (group 2) compared to control mice (group 1), signifying an elevation in the senescence marker P16 expression in PD. GCV-treated PD mice (group 3) exhibited a significant reduction in luminescence intensity compared to PD mice, indicating decreased P16 levels. To validate this effect, RFP expression in gingival tissue was assessed using fluorescence microscopy. The RFP signal was amplified using immunostaining with an anti-RFP antibody. A substantial increase in RFP intensity in the gingiva of PD mice was observed, suggesting an augmentation in p16-positive cells (Figure 1D,E). GCV-treated PD mice exhibited a marked reduction in RFP intensity in the gingiva compared to untreated PD mice, corroborating results observed through luminescence imaging. Furthermore, real-time PCR (Figure 1F) and immunoblotting (Figure 2A) analysis of gingival tissue revealed p16 induction by PD, which was diminished after GCV treatment. These findings highlight the upregulation of P16 expression in PD and the reduction in PD-induced p16-positive cell levels in the P-16 3MR model following GCV treatment.

### 3.2. Elimination of P16-Positive Cells Inhibits Senescence and the Release of SASP Pro-Inflammatory Mediators in PD

The pathogenesis of PD involves a complex interplay between microbial infection, host immune response, and various cellular processes such as inflammation, cellular stress, and DNA damage [22]. Chronic inflammation and cellular stress lead to persistent DNA damage, activating DNA damage response pathways, which are closely interconnected with the mechanisms driving cellular senescence [23]. To further study the molecular mechanisms underlying the relationship between senescence and PD, several markers and key mediators involved in cellular senescence were examined. Western blotting (WB) analysis (Figure 2A,B) revealed that senescence markers p21 and p53, as well as p-H2A.X, a DNA damage and cellular stress marker, were significantly elevated in PD models. This effect was significantly alleviated by GCV treatment. Conversely, the regulator of cell cycle Cyclin D1 expression was reduced in PD and reversed with GCV treatment, as shown in Figure 2A,B. Immunofluorescence staining confirmed the WB findings, showing increased p21 (Figure 2C,D) and endogenous p-H2A.X (Figure 2E,F) levels in PD models, which were reduced following GCV treatment. The senescence-associated secretory phenotype (SASP) is a hallmark of cellular senescence. SASP consists of various factors secreted by senescent cells, including pro-inflammatory cytokines, chemokines, growth factors, and proteases. These secreted factors play a significant role in both the maintenance of the senescent state and the modulation of the tissue microenvironment [24,25]. Several important SASP factors were examined using WB (Figure 2A,B) and qPCR (Figure 2G). SASP pro-inflammatory cytokines IL-6, IL-1β, and TNF-α were significantly increased in PD models. This was associated with equivalent elevated levels of periodontal tissue damaging factors, including MMP9, RANKL, and IL17. Following GCV treatment, these effects were significantly reduced. Collectively, these results suggest that deleting p16-positive cells reduced senescence, inflammatory SASP factor activation, and the release of the resultant bone-resorbing mediators, thereby alleviating senescence-related inflammation in PD.

### 3.3. Cellular Senescence Regulates Gingival Inflammatory Cell Infiltration

**Figure 3:**
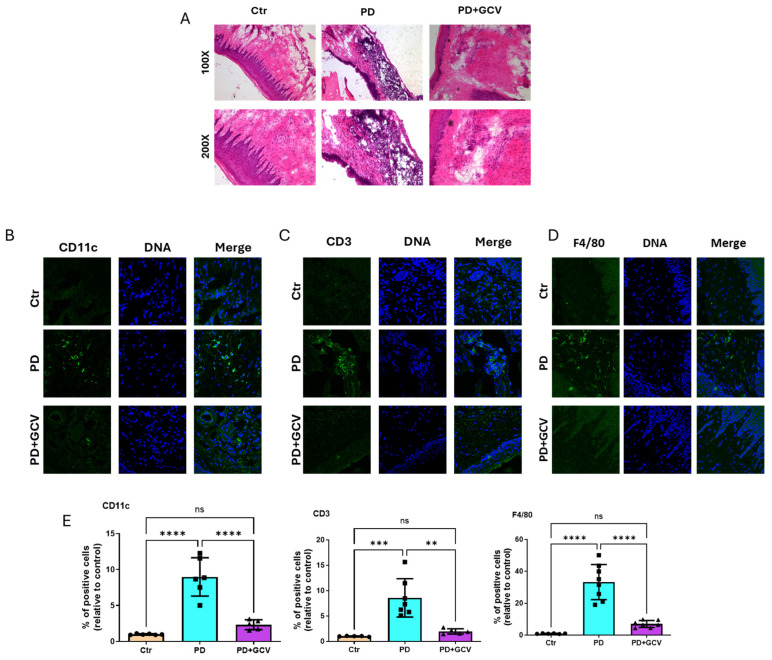
(**A**) Representative hematoxylin–eosin (H&E) staining of gingiva tissue from control, PD, and PD+GCV mice, N = 6–7/group. Top panel: magnification 100× and lower panel: magnification 200×. (**B**) Representative immunofluorescence staining of CD11C in gingiva tissue. Nuclei were stained by Hoechst. (**C**) Representative immunofluorescence staining of CD3 in gingiva tissue. Nuclei were stained by Hoechst. (**D**) Representative immunofluorescence staining of F4/80 in gingiva tissue. Nuclei were stained by Hoechst. (**E**) Quantitation of the CD11C, CD3, and F4/80 from 3B-D using Image J software (NIH) and plotted as relative fold change in positive percentage. N = 5–8/group. ** indicates *p* < 0.01, *** indicates *p* < 0.001, **** indicates *p* < 0.0001, and ns indicates *p* > 0.05.

In PD, disease sites are characterized by infiltration of various inflammatory cells, such as macrophages, dendritic cells (DCs), and T cells, among others [19]. Macrophages are known to play a significant role in the inflammatory processes contributing to bone loss in PD [26]. Additionally, activated DCs have been implicated in directing a bone-damaging T cell response [19]. To illustrate the impact of senescence on PD pathogenesis and progression, we utilized H&E staining to visualize the structural and inflammatory changes in mice gingiva. Analysis revealed the presence of inflammatory infiltrates, connective tissue destruction, and epithelial changes in PD gingival sites compared to the control group. Treatment with GCV alleviated inflammation and tissue damage in mouse gingiva (Figure 3A). Furthermore, immunofluorescence staining indicated an increase in macrophages, DCs, and T cells in the gingival tissue of the PD group, which was notably reduced in the GCV treatment group (Figure 3B–E). These findings further underscore the role of senescence in mediating the recruitment of inflammatory cells and the associated tissue damage in PD.

### 3.4. Osteoclastic Density at PD Sites Is Regulated by Senescence

**Figure 4:**
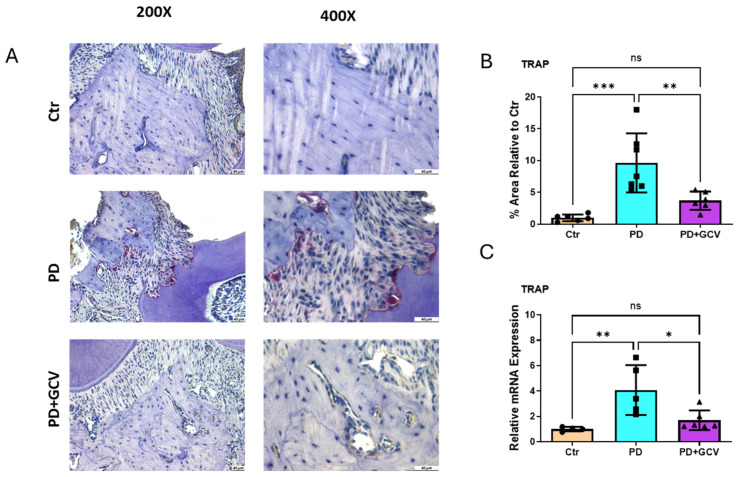
(**A**) Representative TRAP staining of maxillae from control, PD, and PD+GCV mice. TRAP-positive osteoclasts (purple color) were along the bone surface. Left panel: magnification 200× and right panel: magnification 400×. (**B**) Quantitation of TRAP-positive cells using Image J software (NIH) and plotted as relative fold change in positive area. N = 6–7/group. (**C**) SYBR green real-time PCR amplification of TRAP from gingiva tissue. The results shown represent mean ± SEM for 5–7 animals in each group, two replicates. * Indicates *p* < 0.05, ** indicates *p* < 0.01, *** indicates *p* < 0.001, and ns indicates *p* > 0.05.

Upregulated periodontal inflammation promotes osteoclast differentiation and activation. TRAP staining (Figure 4A,B) and mRNA expression (Figure 4C) of alveolar bone tissue showed a significant increase in osteoclastic density with PD. Interestingly, the selective elimination of senescent cells in GCV-treated PD animals significantly reduced TRAP-positive multinucleated cell count in comparison to the PD group without GCV treatment. These results document the bone degenerative role of cellular senescence in PD.

### 3.5. Senescence Inhibition Alleviates Inflammatory Alveolar Bone Loss in PD

**Figure 5:**
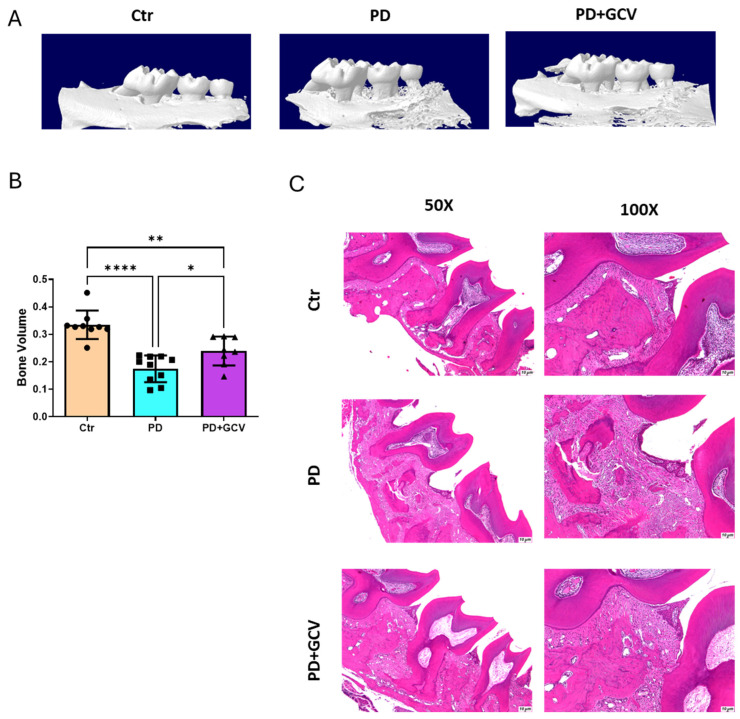
(**A**) Representative 3D micro-CT images of maxillae in the control, PD, and PD+GCV mice. (**B**) Quantitation of bone volume around 2nd molar N = 9–10/group. (**C**) Representative hematoxylin–eosin staining of maxillae from control, PD, and PD+GCV mice, N = 6–7/group. Left panel: magnification 50× and right panel: magnification 100×. * Indicates *p* < 0.05, ** indicates *p* < 0.01, and **** indicates *p* < 0.0001.

To further establish the role of senescence in experimental PD at the causal level, micro-CT scanning was employed to assess the alveolar bone volume. The results shown in Figure 5A,B indicate a significant decrease in bone volume in the PD group, whereas the administration of GCV, aimed at ablating senescent cells, notably mitigated inflammatory bone loss. Histological sections revealed more pronounced alveolar bone destruction in the PD group compared to the control group, a condition that was ameliorated in the GCV-treated PD group (Figure 5C), thus validating the findings of the micro-CT 3D volumetric bone analysis. These findings provide further evidence of cellular senescence as a pivotal process in the inflammatory bone loss observed in PD.

## 4. Discussion

Periodontitis (PD) is recognized as a chronic inflammatory disease that is particularly prevalent among the aging population, a phenomenon often referred to as “inflammaging”. Analysis of the NHANES data from 2009 to 2010 illustrates a clear correlation between age and PD prevalence, with rates increasing from 24.4% in individuals aged 30–34 years to 70.1% in those aged 65 years and older [27]. Furthermore, PD frequently coexists with other more significant age-related diseases (ARDs) [28,29,30,31,32,33], although the precise mechanisms underlying these associations remain to be elucidated. Increased accumulation of senescent cells occurs with advanced age, contributing to the pathophysiology of ARDs. ARDs are characterized by increased peripheral inflammation and a reduction in immune-surveillance capabilities [34,35,36]. Removal of senescent cells reduces ARDs and increases longevity in mice [37]. Despite the established links between senescence and multiple age-related conditions, the specific role of senescence in the development and progression of PD remains unclear. Here, we conducted cause-and-effect studies for the first time utilizing P163MR reporter mice that showed the principal role of cellular senescence in PD pathogenesis.

In this study, experimental PD was induced in a p16-3MR transgenic mice model. This model utilizes the mouse p16^INK4a^ promoter to drive the expression of the 3MR (trimodality reporter) fusion protein [16,17]. Synthetic Renilla luciferase (LUC) enables bioluminescent imaging of P16-positive senescent cells in living animals. Red fluorescent protein-expressing senescent cells can be tracked using fluorescence imaging. Truncated herpes simplex virus 1 (HSV-1) thymidine kinase (HSV-TK) sensitizes cells to ganciclovir (GCV), enabling selective elimination of p16^INK4a^-positive cells. This combination of features facilitates the in vivo identification of p16 activation at the cellular level and the study of the consequences of selectively eliminating p16^INK4a^-positive cells via GCV treatment [16,17].

Our previous studies suggested that PD, when combined with advanced age, leads to immune senescence and alveolar bone loss in wild-type C57/B6 mice. Notably, these effects were ablated by the senolytic agent rapamycin [14,38]. Compared with the off-target and pleiotropic effects of rapamycin [39] or other senolytic agents, the p16-3MR mice model enables the selective targeting of senescent cells in periodontal tissues without affecting healthy cells. In the presented study, it was observed that P16-positive cells increased in oral tissues of p16-3MR transgenic mice with PD, as demonstrated through in vivo bioluminescent and fluorescent RFP imaging (Figure 1). Furthermore, treatment with GCV resulted in the selective ablation of senescent cells. This finding was substantiated by qPCR, Western blot analysis, and immunolabeling of senescence (P16, P21, and P53), cellular stress (p-H2a.X), and cell cycle (Cyclin D1) markers (Figure 1 and Figure 2). These findings justify the feasibility of this model in establishing a causative relationship of senescence with PD.

Senescent cells are resistant to apoptosis, functionally impaired, and express the senescence-associated secretory phenotype (SASP). The SASP consists of secreted pro-inflammatory cytokines (e.g., IL-1b, IL-6, TNF) and extracellular vesicles including exosomes. The pathogenic role of SASP exosomes in inflammaging and age-related diseases has been documented in previous studies [14]. Our research has demonstrated that exosomes derived from periodontal pathogen-infected dendritic cells can transmit senescence to bystander cells and accelerate inflammatory alveolar bone loss in vivo [13,14]. However, the specific mechanisms involved remain unclear, and it is not yet established whether senescence is a causal factor or merely associated with PD. Therefore, cause-and-effect studies in the experimental PD model were conducted to elucidate the role of senescence and the SASP in PD. Here, immunoblotting and mRNA analyses of gingival tissue showed that the selective elimination of p16-positive senescent cells dampened SASP inflammatory cytokine release such as IL1b, IL6, and TNF (Figure 2). In addition, this was associated with downregulation of connective tissue damaging (MMP9) and other bone-resorbing (RANKL and IL17) mediators, demonstrating the role of senescence in periodontal inflammation.

Polymorphonuclear leukocytes are the first responders to periodontal infection and attempt to clear oral microbes [40]. “Frustrated” phagocytosis prompts the release of reactive oxygen species (ROS), collagenases, and other inflammatory meditators, promoting tissue damage [41]. Established PD lesions contain B cells, plasma cells, and macrophages [42,43]. The hallmark of the severe PD lesion is the infiltration of DC clusters with T cells in lamina propria of gingival tissue [44]. Unregulated activation of gingival DCs in situ promotes Th17-mediated bone degeneration [19]. Dysbiotic periodontal pathogen Porphyromonas gingivalis (Pg) invades DCs in the PD lesion and in the bloodstream [40]. The significance of Pg invasion of DCs for immune senescence has only recently been identified by our group in vivo [13,14]. Senescent macrophages have been documented in the gingiva of diabetic mice and were found to be ameliorated by the senolytic agent metformin [30]. In the current study, the deletion of senescent cells led to a decrease in the inflammatory cell infiltrate of PD lesions, including DCs, T cells, and macrophages, as well as a reduction in tissue damage, as evidenced by immunolabeling and histological analysis (Figure 3). This further supports the role of cellular senescence in reprogramming the activation of key immune cells in the pathogenesis of PD.

Bone loss during aging, including alveolar bone loss in the context of PD, is influenced by impaired homeostasis of bone metabolism [45]. The activation of immune cells and the associated inflammatory and bone-resorbing factors released in response to periodontal infection stimulate osteoclastic alveolar bone loss [46]. Chronic inflammation associated with PD also inhibits osteoblast function, leading to impaired bone formation [46,47,48]. Using p16-3MR, we found that clearance of senescent cells in mice subjected to experimental PD alleviates the pathogenesis of PD by regulating Trap-positive bone-resorbing cell activation (Figure 4) and the resultant inflammatory bone loss, as shown by micro-CT scanning (Figure 5).

While this study provides valuable insights into the role of senescence in periodontal disease (PD), some limitations should be acknowledged.

The p16-3MR mouse model, while an invaluable tool for studying cellular senescence, may not fully replicate human PD pathogenesis. Differences in immune system dynamics and senescence pathways between mice and humans may influence the generalizability of our findings. Future studies involving humanized models or clinical samples will be critical for validation.

The use of ganciclovir in this study demonstrated efficacy in ablating senescent cell burden and associated inflammatory factors, thereby enabling a comprehensive examination of the role of senescence in periodontal disease. However, validation with pharmacological inhibition of senescence using other senolytic drugs, such as dasatinib and quercetin, would provide enhanced translational relevance to these findings.

Our focus on key senescence-associated secretory phenotype (SASP) factors, such as IL-6 and IL-1β, provides insights into the inflammatory milieu associated with PD. However, SASP encompasses a broad spectrum of components, including growth factors and extracellular vesicles, which were not explored in this study. These elements could influence disease progression and thus warrant further investigation.

The short experimental timelines employed in this study may not adequately capture the long-term effects of senescence ablation. Longitudinal studies are needed to evaluate the sustainability of therapeutic effects and their impact on disease progression over time.

Oral microbiome and microbial diversity are key players in PD pathogenesis. Variations in oral microbiota or specific bacterial species may influence the outcomes of senescence-targeted therapies; thus, integrating microbiome analyses into future studies will help elucidate the interplay between microbial communities and cellular senescence in PD. Senolytic therapy is an emerging field in biomedicine focused on selectively eliminating senescent cells to improve health and combat age-related diseases [49]. Senescent cells, which have permanently exited the cell cycle in response to stress or damage, accumulate in tissues over time and contribute to aging and various pathologies through their senescence-associated secretory phenotype (SASP). The goal of senolytic therapy is to target and remove these harmful cells, thereby mitigating their negative effects on tissue function and health [50]. Our study suggests that senolytic therapy holds significant therapeutic potential for the treatment of periodontitis by selectively eliminating senescent cells, reducing chronic inflammation, and promoting tissue repair. Further research and clinical trials are needed to establish the safety and efficacy of senolytic therapy in the context of periodontitis. Ongoing studies will help determine optimal dosing, treatment duration, and potential combination therapies.

## Data Availability

The data that support the findings of this study are available on request from the corresponding author.

## Supplementary Materials

The following supporting information can be downloaded at: https://www.mdpi.com/article/10.3390/cells14030226/s1, Figure S1: Genotyping was accomplished by PCR using primer pairs spanning the transgenic region.; Table S1: Primers for genotyping; Table S2: Primers for the realtime-PCR; Table S3: Primary antibodies for Immunoblotting; Table S4: Primary antibodies for Immunofluorescence.

## Abbreviations

The following abbreviations are used in this manuscript:

PD	Periodontitis
EVs	extracellular vesicles
GCV	ganciclovir
DCs	dendritic cells
mRFP	monomeric red fluorescent protein
Pg	Porphyromonas gingivalis
SASP	senescence-associated secretory phenotype
HSV-TK	herpes simplex virus-1 thymidine kinase
mtDNA	mitochondrial DNA
ROS	reactive oxygen species
LCU	synthetic Renilla luciferase
ARDs	age-related diseases
3MR	trimodality reporter
EDTA	ethylenediaminetetraacetic acid
H&E	hematoxylin and eosin
