# Supplementary material for: The Role of Senescence in Experimental Periodontitis at the Causal Level: An in Vivo Study
Source: Cells. 2025 Feb 5;14(3):226. doi: 10.3390/cells14030226 (PMC11817363; doi:10.3390/cells14030226)
Supplement: Supplementary file 1 [file cells-14-00226-s001.zip › cells-3412571-supplementary.pdf]

cells

MDPI

Supplementary Materials

Role of Senescence in Experimental Periodontitis at The Causal Level: An In Vivo Study

Xiaogang Chu, Mahmoud Elashiry, Angelica Carroll, Celine Temothius Cornelius, Christopher W. Cutler and Ranya Elsayed *

Department of Periodontics, Dental College of Georgia, Augusta University, GA 30912, Augusta, Georgia, USA; xichu@augusta.edu (X.C.); melashiry@augusta.edu (M.E.); ancarroll@augusta.edu (A.C.); cconliustimot@augusta.edu (C.T.C.); chcutler@augusta.edu (C.W.C.)
* Correspondence: relsayed@augusta.edu; Tel: 706-446-5176

Supplementary Table S1. Primers for genotyping.

**P16 F**	GATGATGGGCAACGTTCACG
**P16 R**	TTCCCAGCGGTACACAAAGA
**3MR R**	CCAAGCGGTGAGGTACTTGT

Supplementary Table S2. Primers for the realtime-PCR.

**p16 Ink4A**	F: AATCTCCGCGAGGAAAGC
	R: GTCTGCAGCGGACTCCAT
**IL-6**	F: TAGTCCTTCCTACCCCAATTTCC
	R: CGCACTAGGTTTGCCGAGTA
**TNF-α**	F: ATAGCTCCCAGAAAAGCAAGC,
	R: CACCCCGAAGTTCAGTAGACA
**TRAP**	F: CACTCCCACCCTGAGATTTGT
	R: CATCGTCTGCACGGTTCTG
**MMP9**	F: CTGGACAGCCAGACACTAAAG
	R: CTCGCGGCAAGTCTTCAGAG
**RANKL**	F: TGTACTTTCGAGCGCAGATG
	R: CCCACAATGTGTTGCAGTTC
**Actin**	F: CTAAGGCCAACCGTGAAAAG
	R: ACCAGAGGCATACAGGGACA

Supplementary Table S3. Primary antibodies for Immunoblotting.

**Antibody**	**Cat. #**	**Vendor**	**Dilution**
P16	Ab211542	Abcam	1:500
P21	ab188224	Abcam	1:1000
Cyclin D1	2ms-210-p	Neomarkers	1:500
p-H2A.X	05-636	MilliporeSigma	1:1000
P53	Ms-738-p	Neomarkers	1:500
IL1b	12703	Cell signaling	1:1000
IL17	MAB421	R&D Systems	1:500
GAPDH	5174	Cell signaling	1:3000

Supplementary Table S4. Primary antibodies for Immunofluorescence.

Antibody	Cat. #	Vendor	dilution
RFP	600-401-379	ROCKLAND	1:300
p-H2A.X	05-636	MilliporeSigma	1:200
P21	ab188224	Abcam	1:100
CD3	14-0031	Invitrogen	1:100
CD11C	14-0114	Invitrogen	1:100
F4/80	14-4801	Invitrogen	1:100

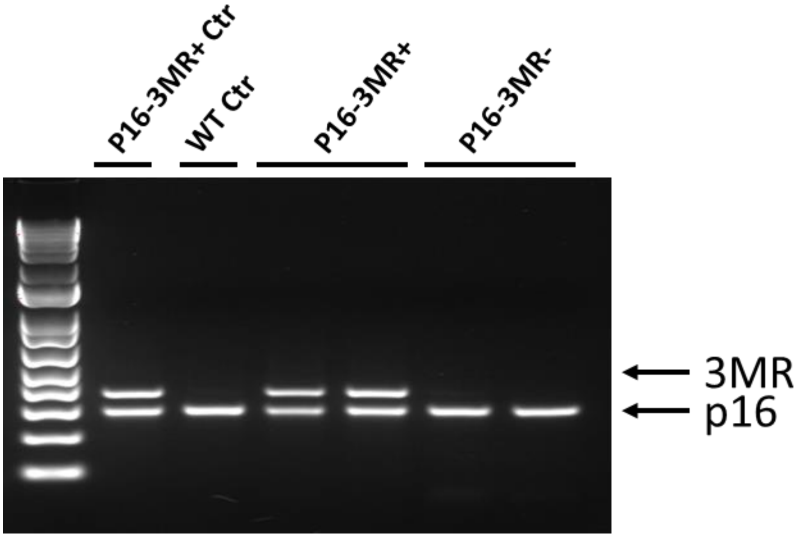

Supplementary Figure S1. Genotyping was accomplished by PCR using primer pairs spanning the transgenic region. Genotype patterns of the polymorphisms displayed by 2% agarose gel electrophoresis. M, 100 bp DNA ladder. The p16-3MR positive shows 2 bands and negative (WT) shows one band.
